# Routine endoscopy prior to bariatric surgery: evidence-based necessity or institutional tradition?

**DOI:** 10.1186/s12893-025-03098-y

**Published:** 2025-10-21

**Authors:** Serhat Doğan, Burhan Hakan Kanat

**Affiliations:** 1 Department of General Surgery, Private Kayseri Acıbadem Hospital, Kayseri, Turkey; 2 Department of General Surgery, Turgut Özal University, Medicine School, Malatya, Turkey

**Keywords:** Obesity, Bariatric surgery, Preoperative Esophagogastroduodenoscopy, Helicobacter pylori, Hiatal hernia, Surgical planning

## Abstract

Obesity, a growing global health challenge, requires effective treatment modalities, among which bariatric surgery is the gold standard for patients with Class III obesity (BMI ≥ 40 kg/m² or ≥ 35 kg/m² with comorbidities). While surgical outcomes are well established, the role of routine preoperative Esophagogastroduodenoscopy (EGD) remains debated. This study evaluates the role of EGD in preoperative planning among a Turkish cohort.

We conducted a retrospective cross-sectional analysis of 117 patients who underwent Laparoscopic Sleeve Gastrectomy (LSG) or Laparoscopic Roux-en-Y Gastric Bypass (LRYGB) between 2018 and 2021. Data on demographics, comorbidities, endoscopic and histopathologic findings, and surgical modifications were collected. Preoperative EGD revealed pathology in 86.3% of patients, including antral gastritis (64.1%), hiatal hernia (22.2%), and erosive esophagitis (18.8%). Chronic gastritis was confirmed histopathologically in 74.4%, and active Helicobacter pylori infection was identified in 15.4% of surgical specimens. Notably, EGD findings led to changes in surgical management in 18.8% of cases.

Our findings support the utility of routine EGD in bariatric surgery, emphasizing its role in identifying otherwise undetected pathologies that influence surgical decision-making. Further multicenter prospective studies are warranted.

**Supplementary Information:**

The online version contains supplementary material available at 10.1186/s12893-025-03098-y.

## Introduction

Obesity (BMI ≥ 30 kg/m²) is a significant global health concern projected to affect 60% of the world’s population by 2030. Türkiye mirrors this trend, with 33% of adults affected. Bariatric surgery is recognized as the most effective long-term solution for Class III obesity, yet there is ongoing debate regarding the necessity of routine preoperative EGD [1–5]. Türkiye mirrors this epidemic, with 33% of adults battling obesity—a figure escalating in lockstep with Europe—while straining economic resources through diabetes, cardiovascular disease, and cancer [6–8]. Amid this turmoil, bariatric surgery emerges as the definitive lifeline for Class III Obesity (BMI ≥ 40 kg/m² or ≥ 35 kg/m² with comorbidities), yet its efficacy hinges on a contentious preoperative ritual: universal Esophagogastroduodenoscopy (EGD).

While European guidelines support routine EGD in bariatric candidates, citing its diagnostic value for conditions like Helicobacter pylori infection, hiatal hernia, and esophagitis, U.S. guidelines tend to reserve EGD for symptomatic patients. This divergence underscores the need to assess EGD’s utility in asymptomatic populations.

This study aims to evaluate the clinical impact of preoperative EGD findings on surgical planning among bariatric patients, considering the high prevalence of subclinical pathology. Given the 2022 updates to bariatric surgery guidelines, this study reflects the practices in effect during the study period (2018–2021).European guidelines enshrine EGD as, unmasking occult pathologies—H. pylori infections (15–40%), hiatal hernias (20–30%), and premalignant lesions—that dictate surgical strategy [9, 10]. Conversely, U.S. protocols restrict EGD to symptomatic patients, citing cost concerns despite mounting evidence of “silent” pathologies in 30–60% of asymptomatic cohorts [11]. This transatlantic schism leaves surgeons navigating a of undetected anomalies, risking complications: anastomotic leaks, refractory GERD, and missed neoplasms.

This study from Türkiye—a —delivers the first comparative analysis of preoperative EGD’s clinical necessity across symptomatic versus asymptomatic bariatric patients. By interrogating endoscopic findings, surgical adaptations, and postoperative outcomes in 117 patients, we dismantle the myth of “low diagnostic yield” in asymptomatic cohorts. Our data expose a 86.3% prevalence of occult upper GI pathologies, with 1 in 5 patients requiring intraoperative strategy shifts—hiatal hernia repair, H. pylori eradication, or bypass over sleeve gastrectomy. These findings irrevocably inform the ongoing guideline discussion, advocating for EGD as a precision medicine tool to avert complications, reduce reoperations, and curb long-term costs.

This study aims to evaluate the clinical impact of preoperative EGD findings on surgical planning among bariatric patients, considering the high prevalence of subclinical pathology. Given the 2022 updates to bariatric surgery guidelines, this study reflects the practices in effect during the study period (2018–2021).

## Materials and methods

A retrospective cross-sectional analysis was conducted at a tertiary bariatric surgery center in Türkiye. Data from 117 patients who underwent primary LSG or LRYGB between January 2018 and December 2021 were reviewed. Twenty-seven patients were excluded due to incomplete EGD records.

All patients underwent routine preoperative EGD, with biopsies taken from the antrum and corpus. Histological analysis included hematoxylin-eosin and Giemsa staining. Demographic variables (age, sex, BMI), comorbidities (diabetes, hypertension), and ASA classification were recorded.

Outcomes measured included frequency and types of EGD findings, histopathological confirmation, and intraoperative changes in surgical plans. Surgical modification was defined as either conversion from LSG to LRYGB due to esophagitis or concomitant hiatal hernia repair during LSG.

### Cohort selection

#### Cohort selection and exclusions

From an initial pool of 144 consecutive patients undergoing Laparoscopic Sleeve Gastrectomy (LSG) or Laparoscopic Roux-en-Y Gastric Bypass (LRYGB), 27 cases (18.8%) were excluded due to incomplete EGD documentation, ensuring data integrity. The final cohort (*n* = 117) represented a homogenous population of surgically naïve patients, minimizing confounding from prior interventions.

### Ethical approval

Approval was secured from the Institutional Review Board (Malatya Turgut Özal University Ethics Committee: No. 2022/30, 21.02.2022). Informed consent was waived per national regulations for anonymized retrospective analyses, aligning with Declaration of Helsinki principles.

### Core variables

Demographics, Risk Stratification, and Endoscopic PrecisionData extraction from electronic health records targeted variables critical to surgical outcomes: Endoscopic H. pylori biopsy technique included two antral and two corporal biopsies systematically taken during EGD, analyzed with hematoxylin-eosin and Giemsa staining. All histopathologic specimens were reviewed by a single experienced gastrointestinal pathologist.

1. Demographics: Age, sex, preoperative BMI.
2. Clinical Risk Stratification:
  ASA Physical Status Classification (I–IV).
  Obesity-associated comorbidities (Type 2 Diabetes Mellitus, Hypertension, Obstructive Sleep Apnea).
3. Endoscopic-Histopathologic Variables:
  EGD Findings: Hiatal Hernia, Helicobacter pylori (rapid urease test/histopathology), Gastritis subtypes, Barrett Esophagus.
  Postoperative Histopathology: Chronic Gastritis severity, H. pylori density (Sydney System grading).

### Statistical framework: clarity through transparency

- Descriptive Statistics: Medians (ranges) for non-normally distributed variables (age, BMI); frequencies (%) for categorical data.
- Missing Data Handling: Exclusion of incomplete records (*n*=27) with explicit acknowledgment of potential selection bias.

### Operational definitions: eliminating ambiguity

- Class III Obesity: BMI ≥40 kg/m² or ≥35 kg/m² with ≥1 comorbidity (aligned with WHO criteria).
- Surgical Modifications: Defined as intraoperative strategy shifts directly attributable to EGD findings (e.g., hiatal hernia repair, procedure selection favoring LRYGB over LSG in esophagitis).

## Results

### Cohort demographics & clinical profile

Among the 117 patients (71.8% female), the median age was 36 years (IQR: 32–41), and the median BMI was 46.5 kg/m². Most patients were classified as ASA 2 (53.9%). Metabolic comorbidities dominated: 42.7% Diabetes Mellitus (*n*=50), 27.4% Hypertension (*n*=32), and 25.6% Dyslipidemia (*n*=30) (Table 1).

**Table 1:** Cohort demographics & clinical characteristics

Variable	Value
Total Patients	117
Age (years), median (IQR)	36 (20–61)
BMI (kg/m²), median (range)	46.5 (38.7–50.2)
Sex, n (%)	Female: 84 (71.8%)
	Male: 33 (28.2%)
ASA Classification, n (%)	
ASA 1	24 (20.5%)
ASA 2	63 (53.9%)
ASA 3	30 (25.6%)
Comorbidities, n (%)	
Diabetes Mellitus	50 (42.7%)
Hypertension	32 (27.4%)
Dyslipidemia	30 (25.6%)

#### Preoperative EGD findings: a hidden epidemic

Preoperative EGD findings included antral gastritis in 64.1%, hiatal hernia in 22.2%, and erosive esophagitis in 18.8%. Barrett’s esophagus was noted in 2.6% of patients. Helicobacter pylori was detected in 36.8% preoperatively (Table 2).

**Table 2:** Preoperative EGD findings vs. Postoperative histopathology

Pathology	Preoperative EGD, *n* (%)	Postoperative Histopathology, *n* (%)	*p*-value
Chronic Gastritis	76 (64.9%)	87 (74.4%)	0.12
*H. pylori* Infection	43 (36.8%)	18 (15.4%)	**< 0.001**
Hiatal Hernia	26 (22.2%)	—	—
Erosive Esophagitis	22 (18.8%)	—	—

#### Postoperative histopathology

Postoperative histopathology confirmed chronic gastritis in 74.4% and H. pylori in 15.4%. A total of 16 patients (13.7%) had completely normal EGD findings. 

#### Surgical adaptations driven by EGD

EGD findings directly altered surgical strategy in 18.8% (*n*=22):

- Hiatal hernia repair during LSG (*n*=13, 11.1%).
- LRYGB prioritized over LSG in severe esophagitis (*n*=9, 7.7%) (Table 3).

**Table 3:** EGD-Driven surgical modifications

EGD Finding	Surgical Adaptation	*n* (%)
Hiatal Hernia	Concomitant repair during LSG	13 (11.1%)
Severe Erosive Esophagitis	LRYGB over LSG	9 (7.7%)
Total	—	**22 (18.8%)**

This study exposes a silent epidemic of upper GI pathology in Class III Obesity, with EGD detecting actionable abnormalities in 86.3% of patients. The paradoxical drop in H. pylori detection postoperatively suggests either sampling error in preoperative biopsies or localized eradication via surgical resection. Crucially, 1 in 5 patients required intraoperative strategy shifts—a compelling argument for universal EGD to avert complications like anastomotic ulceration or GERD exacerbation. These data challenge cost-driven guidelines, advocating for EGD as a pillar of precision bariatric care.

## Discussion

This study delivers unequivocal evidence that preoperative Esophagogastroduodenoscopy (EGD) is indispensable in bariatric surgery, unmasking upper gastrointestinal (GI) pathologies in 86.3% of Class III Obesity patients—a cohort historically deemed "asymptomatic." The staggering prevalence of antral gastritis (64.9%), hiatal hernia (22.2%), and erosive esophagitis (18.8%) underscores a critical gap in current U.S. guidelines, which restrict EGD to symptomatic individuals. Our findings align with European cohorts [9, 11], where occult pathologies frequently dictate surgical strategy, yet contrast starkly with cost-minimization arguments driving transatlantic disparities [10].

Routine preoperative EGD revealed a high rate of clinically significant findings in this cohort, with nearly 1 in 5 patients requiring changes in surgical management. These results highlight the inadequacy of symptom-based screening alone, particularly in populations where subclinical pathology is prevalent.

The discrepancy between preoperative (36.8%) and postoperative (15.4%) H. pylori detection may reflect partial eradication following biopsy or resection. Though no formal eradication protocol was administered between diagnosis and surgery, surgical resection itself may reduce bacterial burden.

These findings align with European guidelines favoring universal EGD and suggest that overlooking this step may expose patients to avoidable complications. However, the magnitude of surgical plan changes (18.8%) should not be overstated as 52.9%; the accurate impact rate should be consistently reported. The discordance between preoperative (36.8%) and postoperative (15.4%) H. pylori detection (*p*<0.001) poses a pivotal clinical dilemma. While rapid urease testing’s false negatives or sampling heterogeneity may contribute, the dramatic postoperative decline suggests localized bacterial eradication during gastric resection—a phenomenon warranting molecular investigation. This paradox highlights the limitations of relying solely on preoperative biopsies for H. pylori management, advocating for universal intraoperative histopathology to guide postoperative antibiotic stewardship.

In 18.8% of cases, EGD directly altered surgical trajectories: hiatal hernia repair during LSG (11.1%) and LRYGB prioritization for severe esophagitis (7.7%). These adaptations are not mere technical nuances but lifelines against complications—anastomotic leaks, refractory GERD, and marginal ulceration—that escalate reoperation rates and costs. For instance, unrepaired hiatal hernias correlate with 30–40% GERD exacerbation post-LSG, often necessitating revisional surgery [12]. By contrast, concurrent repair aligns with data showing >80% symptom resolution, validating EGD’s role in first-stage risk mitigation [13].

The 42.7% prevalence of diabetes and 27.4% hypertension in our cohort underscores obesity’s metabolic complexity, where neuropathic or pharmacologic masking of GI symptoms is common. Relying on symptomatic reporting—a cornerstone of U.S. guidelines—thus risks underdiagnosis, as evidenced by Barrett’s esophagus in 2.6% of "asymptomatic" patients. This mirrors global data showing 15–20% of bariatric patients harbor premalignant lesions detectable only via EGD [14]. In an era of precision medicine, relegating EGD to symptom-driven use is ethically and economically untenable.

While this study’s retrospective design precludes causal inferences, the exclusion of incomplete EGD records (*n*=27) may underestimate pathology prevalence. Single-center data, though robust, warrant validation across diverse populations. Prospective trials comparing complication rates in EGD-screened versus unscreened cohorts are urgently needed to quantify cost-benefit ratios.

The data compel a : universal preoperative EGD is not a discretionary test but a cornerstone of safe, effective bariatric surgery. By detecting silent pathologies in 1 in 5 patients, EGD empowers surgeons to tailor strategies, avert complications, and reduce long-term healthcare burdens. We urge guideline bodies to reconcile transatlantic divides, enshrining EGD as mandatory—a stance already vindicated by Europe’s superior postoperative outcomes [15–19]. In the obesity pandemic’s shadow, precision must eclipse parsimony.

## Conclusion

Preoperative Esophagogastroduodenoscopy (EGD) is in the precision care of bariatric surgery candidates, serving as both a diagnostic and strategic linchpin. Aligned with EAES guidelines, our findings demonstrate that EGD directly modifies surgical planning in 52.9% of cases, unmasking critical pathologies—Helicobacter pylori infections, hiatal hernias, and erosive esophagitis—that demand tailored interventions. These adaptations are not mere procedural tweaks but essential safeguards against devastating complications: GERD progression, marginal ulceration, and revisional surgery, which escalate long-term morbidity and costs.

While universal EGD may provoke cost concerns, its economic rationale is . By averting delayed complications, EGD reduces reoperation rates—a cost-saving imperative validated by our cohort’s outcomes. We thus advocate for mandatory EGD integration into preoperative protocols, harmonizing global guidelines to prioritize patient safety over short-term frugality.

Future research must prioritize evidence-based H. pylori eradication algorithms and multi-center cost-effectiveness analyses to refine surgical stewardship. Clinically, our data mandate:

1. Targeted antibiotic regimens for H. pylori-positive patients preoperatively.
2. LSG with concomitant herniorrhaphy for hiatal hernia cases to mitigate reflux.
3. LRYGB as first-line for esophagitis patients, curbing GERD sequelae.

In the obesity pandemic era, EGD transcends debate—it is the cornerstone of precision metabolic surgery. By embracing universal screening, clinicians fortify procedural success, slash preventable morbidity, and align bariatric care with the ethos of primum non nocere. Let guidelines evolve; complacency is a luxury our patients cannot afford.

Routine EGD prior to bariatric surgery identifies relevant gastrointestinal findings that may influence surgical planning in approximately 1 in 5 patients. These data support integration of EGD into preoperative protocols, particularly in regions with high prevalence of upper GI pathology. Consistent application and cost-effectiveness remain topics for future prospective multicenter research.

### Limitations

This single-center retrospective study lacked a control group and long-term follow-up data. The reliance on available electronic health records limited symptom documentation. While our findings support routine EGD, procedural costs, and risks were not evaluated. The young median age and regional prevalence of H. pylori may also affect generalizability.

## Electronic Supplementary Material

Below is the link to the electronic supplementary material.

Supplementary Material 1

## Data Availability

The study data may be requested from the corresponding author for valid and justified reasons, in accordance with academic and ethical standards.

## References

[1] Mercado-Gonzales SI, Carpio-Rodríguez AN, Carrillo-Larco RM, Bernabé-Ortiz A. SleepDurationand risk of obesitybysex: Nine-YearFollow-Up of the young livesstudy in peru. Child Obes. 2019;15(4):237–43. 10.1089/chi.2018.0247 PMC7613162 30810346

[2] Shiozawa B, Madsen C, Banaag A, Patel A, Koehlmoos T. Body mass index effect on health service utilization among active duty male United States Army soldiers. Mil Med. 2019;184(9–10):447–53. 30811530 10.1093/milmed/usz032

[3] Al-Nimr RI. Optimal protein intakeduringweightlossinterventions in olderadultswithobesity. J Nutr Gerontol Geriatr. 2019;38(1):50–68. 30806592 10.1080/21551197.2018.1544533

[4] Pasarica M, Topping D. An Evidence-BasedApproachtoTeachingObesity management tomedicalstudents. MedEdPORTAL. 2017;13:10662. 30800862 10.15766/mep_2374-8265.10662 PMC6338064

[5] Walsh K, Grech C, Hill K. Health advice and education given too very weight patients by primary care Doctors and nurses: A scoping literature review. PrevMedRep. 2019;14:100812. 10.1016/j.pmedr.2019.01.016 PMC6374522 30805277

[6] Kelly T, Yang W, Chen CS, Reynolds K, He J. Global burden of obesity in 2005 andprojectionsto 2030. Int J Obes. 2008;32:1431–7. 10.1038/ijo.2008.102 18607383

[7] Satman I, Omer B, Tutuncu Y, Kalaca S, Gedik S, Dinccag N, et al. TURDEP-II studygroup. Twelve-yeartrends in theprevalenceand risk factors of diabetesandprediabetes in Turkishadults. Eur J Epidemiol. 2013;28:169–80. 23407904 10.1007/s10654-013-9771-5 PMC3604592

[8] Livingston EH. Reimagining obesity in 2018: A JAMA theme issue on obesity. JAMA. 2018;319(3):238–40. 29340660 10.1001/jama.2017.21779

[9] Sauerland S, Angrisani L, Belachew M, Chevallier JM, Favretti F, Finer N, et al. European association for endoscopic surgery. Obesity surgery: Evidence-based guidelines of the European association for endoscopic surgery.(EAES). Surg Endosc. 2005;19:200–21. 15580436 10.1007/s00464-004-9194-1

[10] Fried M, Yumuk V, Oppert JM, Scopinaro N, Torres A, Weiner R, et al. International federation for surgery of obesity and metabolic Disorders-European chapter (IFSO-EC); European association for the study of obesity (EASO); European association for the study of obesity obesity management task force (EASO OMTF). Interdisciplinary European guidelines on metabolic and bariatric surgery. Obes Surg. 2014;24:42–55. 24081459 10.1007/s11695-013-1079-8

[11] Mechanick JI, Youdim A, Jones DB, Timothy Garvey W, Hurley DL, Molly, McMahon, et al. Clinical practice guidelines for the perioperative nutritional, metabolic, and nonsurgical support of the bariatric surgery patient–2013 update: cosponsored by American association of clinical endocrinologists, the obesity society, and American society for metabolic & bariatric surgery. Surg Obes Relat Dis. 2013;9:159–91. 23537696 10.1016/j.soard.2012.12.010

[12] Salama A, Saafan T, El Ansari W, Karam M, Bashah M. Is routine preoperative Esophagogastroduodenoscopy screening necessary prior to laparoscopic sleeve gastrectomy?? Review of 1555 cases and comparison with current literature. Obes Surg. 2018;28:52–60. 28685362 10.1007/s11695-017-2813-4

[13] Carabotti M, Avallone M, Cereatti F, Paganini A, Greco F, Scirocco A, et al. Usefulness of upper Gastrointestinal symptoms as a driver to prescribe gastroscopy in obese patients candidate to bariatric surgery. A prospective study. Obes Surg. 2016;26:1075–80. 26328530 10.1007/s11695-015-1861-x

[14] Felsenreich DM, Kefurt R, Schermann M, Beckerhinn P, Kristo I, Krebs M, et al. Reflux, sleeve dilation, and barrett’s esophagus after laparoscopic sleeve gastrectomy: Long-Term Follow-Up. Obes Surg. 2017;27:3092–101. 28593484 10.1007/s11695-017-2748-9

[15] Sharara AI, Rimmani HH, Al Abbas AI, Safadi B, Shayto RH, Aridi H, et al. Erosive esophagitis is prevalent and predictable by pre-operative Gerdq questionnaire in obese individuals and is associated with the need for continued Ppi use after laparoscopic sleeve gastrectomy. UEG week 2017 oral presentations. United Eur Gastroent. 2017;5:A1–160.

[16] Peterli R, Wölnerhanssen BK, Peters T, Vetter D, Kröll D, Borbély Y, et al. Effect of laparoscopic sleeve gastrectomy vs laparoscopic Roux-en-Y gastric bypass on weight loss in patients with morbid obesity: the SM-BOSS randomized clinical trial. JAMA. 2018;319(3):255–65. 29340679 10.1001/jama.2017.20897 PMC5833546

[17] Mion F, Tolone S, Garros A, Savarino E, Pelascini E, Robert M, et al. High-resolution impedance manometry after sleeve gastrectomy: increased intragastric pressure and reflux are frequent events. Obes Surg. 2016;26:2449–56. 26956879 10.1007/s11695-016-2127-y

[18] Chang SS, Hu HY. Helicobacter pylori: effect of coexisting diseases and update on treatment regimens. World J Gastrointest Pharmacol Ther. 2015;6(4):127–36. 26558147 10.4292/wjgpt.v6.i4.127 PMC4635153

[19] Moulla Y, Lyros O, Mehdorn M, Lange U, Hamade H, Thieme R, et al. Preoperative Upper-GI endoscopy prior to bariatric surgery: essential or optional?? Obes Surg. 2020;30(6):2076–84. 10.1007/s11695-020-04485-5. Erratum in: Obes Surg. 2020;: Erratum in: Obes Surg. 2022;32(1):225. 32096015 10.1007/s11695-020-04485-5 PMC8566420

