# Supplementary material for: Routine endoscopy prior to bariatric surgery: evidence-based necessity or institutional tradition?
Source: BMC Surg. 2025 Oct 21;25:493. doi: 10.1186/s12893-025-03098-y (PMC12542399; doi:10.1186/s12893-025-03098-y)
Supplement: Supplementary file 1 — Supplementary Material 1 [file 12893_2025_3098_MOESM1_ESM.docx]

İ want to ad q7 but can not make ıt

Retrospective study; individual consent for publication was waived due to anonymized data
